# Integrated Transcriptome and Metabolome Analysis Reveals the Regulatory Mechanisms of *FASN* in *Geese* Granulosa Cells

**DOI:** 10.3390/ijms232314717

**Published:** 2022-11-25

**Authors:** Xi Chen, Kailiang Huang, Shenqiang Hu, Gang Lan, Xiang Gan, Shanyan Gao, Yan Deng, Jiwei Hu, Liang Li, Bo Hu, Hua He, Hehe Liu, Lu Xia, Jiwen Wang

**Affiliations:** 1 Farm Animal Genetic Resources Exploration and Innovation Key Laboratory of Sichuan Province, College of Animal Science and Technology, Sichuan Agricultural University, Chengdu 611130, China; 2 Key Laboratory of Agricultural Information Engineering of Sichuan Province, College of Information Engineering, Sichuan Agricultural University, Yaan 625014, China

**Keywords:** *FASN*, granulosa cells, *TLR3*, 5-HT, neuroactive ligand-receptor interaction pathway

## Abstract

*FASN* plays a critical role in lipid metabolism, which is involved in regulating ovarian follicular development. However, the molecular mechanisms of how *FASN* regulate the function of ovarian follicular cells still remain elusive. In this study, by overexpression or interference of *FASN* in pre-hierarchical follicle granulosa cells (phGCs) and hierarchical follicle granulosa cells (hGCs), we analyzed their effects on the granulosa cell transcriptome and metabolome profiles using RNA-Seq and LC-MS/MS, respectively. The results showed that overexpression of *FASN* promoted proinflammatory factors expression by activating *TLR3*/*IRF7* and *TLR3*/*NF-κB* pathways in phGCs, but only by activating *TLR3*/*IRF7* pathways in hGCs. Then, necroptosis and apoptosis were triggered through the *JAK*/*STAT1* pathway (induced by inflammatory factors) and *BAK*/*caspase-7* pathway, respectively. The combined analysis of the metabolome and transcriptome revealed that *FASN* affected the demand of GCs for 5-hydroxytryptamine (5-HT) by activating the neuroactive ligand-receptor interaction pathway in two categorized GCs and only altering the metabolic pathway of tryptophan in phGCs, and ultimately participated in regulating the physiological function of *geese* GCs. Taken together, this study showed that the mechanisms of *FASN* regulating the physiological function of *geese* phGCs and hGCs were similar, but they also had some different characteristics.

## 1. Introduction

As the critical component of ovarian follicles, GCs play an important role in maintaining follicular development. Most of the previous studies have focused on the apoptosis and steroidogenesis of GCs, but less attention has been paid to the de novo lipogenesis (DNL) process in GCs. As a committed step in the process of lipid metabolism, DNL plays an important role in various physiological processes in cells. DNL in mammalian GCs has been a research hotspot over recent years, but the study of DNL in avian GCs is very limited compared with mammals. However, DNL in avian GCs may be more complex because the follicular maturation is accompanied by the accumulation of yolks, which requires a large amount of lipids. Our previous studies demonstrated that fatty acid synthase (*FASN*), a key rate-limiting enzyme in DNL, showed a developmental-stage dependent expression pattern in the GCs of *geese* with different sized follicles [1]. Therefore, we hypothesize that *FASN*-mediated lipid metabolism may be involved in the regulation of *geese* follicular development.

*FASN* catalyzes the formation of palmitic acid, which is a kind of saturated fatty acids (SFA), from acetyl-*CoA* and malonyl-*CoA* in the presence of coenzyme Ⅱ reduced tetrasodium salt (*NADPH*). Several lines of evidence have proven that *FASN* or palmitic acid participate in the regulation of GC function. In *bovine* GCs cultured in vitro, the inhibition of *FASN* by C75 (an inhibitor of *FASN*) reduced progesterone (P_4_) biosynthesis through inhibiting the cholesterol biosynthesis and repressing the cell proliferation through downregulating the expression of *cyclin-D2* [2]. In addition, it was reported that the addition of 150, 300, or 500 mM of palmitic acid could inhibit the *bovine* GC proliferation through inducing apoptosis, and stimulated the biosynthesis of oestradiol-17β (E_2_) by stimulating ovarian *NADPH* dependent enzymes such as the aromatase enzyme complex [3]. Otherwise, palmitic acid was reported to induce the apoptosis of *human* GCs due to its lipotoxicity [4]. Moreover, our recent research suggests that *FASN* interference inhibited P_4_ biosynthesis of *geese* GCs through repressing the expression of steroidogenic acute regulatory protein (*STAR*), while *FASN* overexpression promoted their apoptosis due to the lipotoxicity of the product of *FASN* [5]. The mechanisms, however, of how *FASN* regulates *geese* GC physiological function remain largely unknown.

Transcriptomics is an important means to study gene expression at the RNA level [6]; as an extension of it, metabolomics reveals the mechanism of its participation in life activities through the changes in small molecule metabolites. There have been many reports of using transcriptome or metabolome independently to study GC function, however, the application of combined transcriptome and metabolome techniques in avian GCs has not been reported thus far.

The present study investigated the metabolome and mRNA transcriptome profiling of *FASN*-overexpressed and *FASN*-interfered *geese* GCs through LC-MS/MS and RNA-Seq, respectively for the first time. This comprehensive analysis is expected to elucidate the similarities and differences of the underlying mechanisms by which *FASN* regulates phGC and hGC function, and also identifies the critical metabolites in these mentioned regulations.

## 2. Results

### 2.1. Efficiency of FASN Overexpression and Interference

In order to determine the efficiency of transfection, *pEGFP-N1-FASN*, *pEGFP-N1*, siRNA-*FASN*, and siRNA-NC were transfected into phGCs and hGCs, respectively. Then, the efficiency of *FASN* overexpression and interference were verified by qRT-PCR. For the overexpression group, the expression levels of *FASN* increased to almost 78- and 330-fold compared to the control in phGCs and hGCs, respectively. For the interference group, the expression levels of *FASN* decreased to 0.42- and 0.37-fold compared to the control in phGCs and hGCs, respectively [5].

### 2.2. Metabolomics Analysis

After quality control, 1700 kinds of metabolites in total were obtained from 48 detected GC samples by using LC-MS/MS. In this study, 63 and 54 metabolites were identified in *FASN*-overexpressed phGCs and hGCs, respectively. Thirty-six and 35 metabolites were identified in the *FASN*-interfered phGCs and hGCs, respectively (Table 1). The corresponding quantitative data concerning these identified metabolites are summarized in Table S1.

To explore the potential metabolic pathways that were affected by *FASN*, the roles of the differential metabolites were determined by KEGG pathway enrichment analysis using the hypergeometric test (Table 2). Eight, 13, two, and 23 KEGG pathways were enriched by differential metabolites in these four groups, respectively (Table S2). Pyrimidine metabolism and the amino sugar and nucleotide sugar metabolism pathways were significantly enriched by the differential metabolites in *FASN*-overexpressed phGCs. However, there was no significantly enriched KEGG pathway in the *FASN*-overexpressed hGCs. The biotin metabolism pathway was significantly enriched by the differential metabolites in the *FASN*-interfered phGCs. Serotonergic synapse, neuroactive ligand-receptor interaction, gap junction, taste transduction, inflammatory mediator regulation of TRP channels, cAMP signaling pathway, synaptic vesicle cycle, *PPAR* signaling pathway, adrenergic signaling in cardiomyocytes, vascular smooth muscle contraction, renin secretion, and salivary secretion pathways were significantly enriched by the differential metabolites in the *FASN*-interfered hGCs.

### 2.3. Expression Profile of DEGs

**Figure 1:**
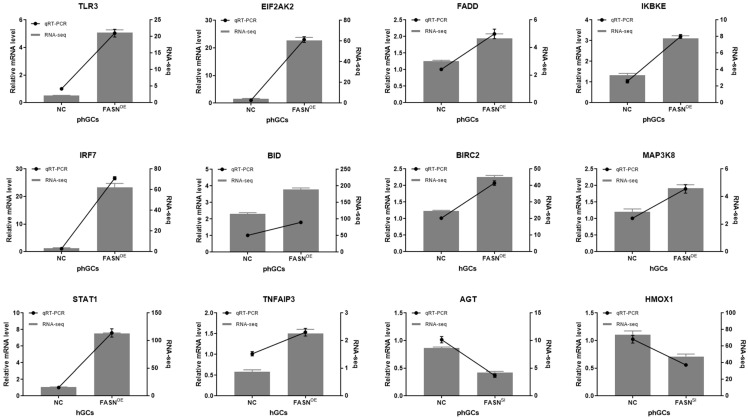
Validation analysis of twelve DEGs by qRT-PCR (line) and RNA-Seq (bar). OE is the abbreviation for overexpression. The expression of genes detected by qRT-PCR were normalized by *β-actin* and the control was set as one. Results were presented as the mean ± SEM of four samples.

To reveal the transcriptional changes caused by the *FASN* overexpression or interference in *geese* GCs, RNA-Seq analysis was performed. The results showed that after *FASN* overexpression, 1099 and 591 DEGs were identified in the phGCs and hGCs, respectively. After *FASN* interference, 224 and 161 DEGs were identified in the phGCs and hGCs, respectively (Table 3). These results demonstrate that a higher transcriptome diversification was presented when performing *FASN* overexpression rather than interference. To verify the accuracy of RNA-Seq, twelve genes were selected for qRT-PCR and the results showed similar expression patterns to RNA-Seq for these genes (Figure 1).

### 2.4. Functional Enrichment Analysis of DEGs

To identify the effects of *FASN* on *geese* GC function, all DEGs were selected for GO enrichment and the KEGG pathway analysis. The first 20 GO terms and KEGG pathways enriched by DEGs in the four groups are listed in Table S3 and Table S4, respectively.

**Figure 2:**
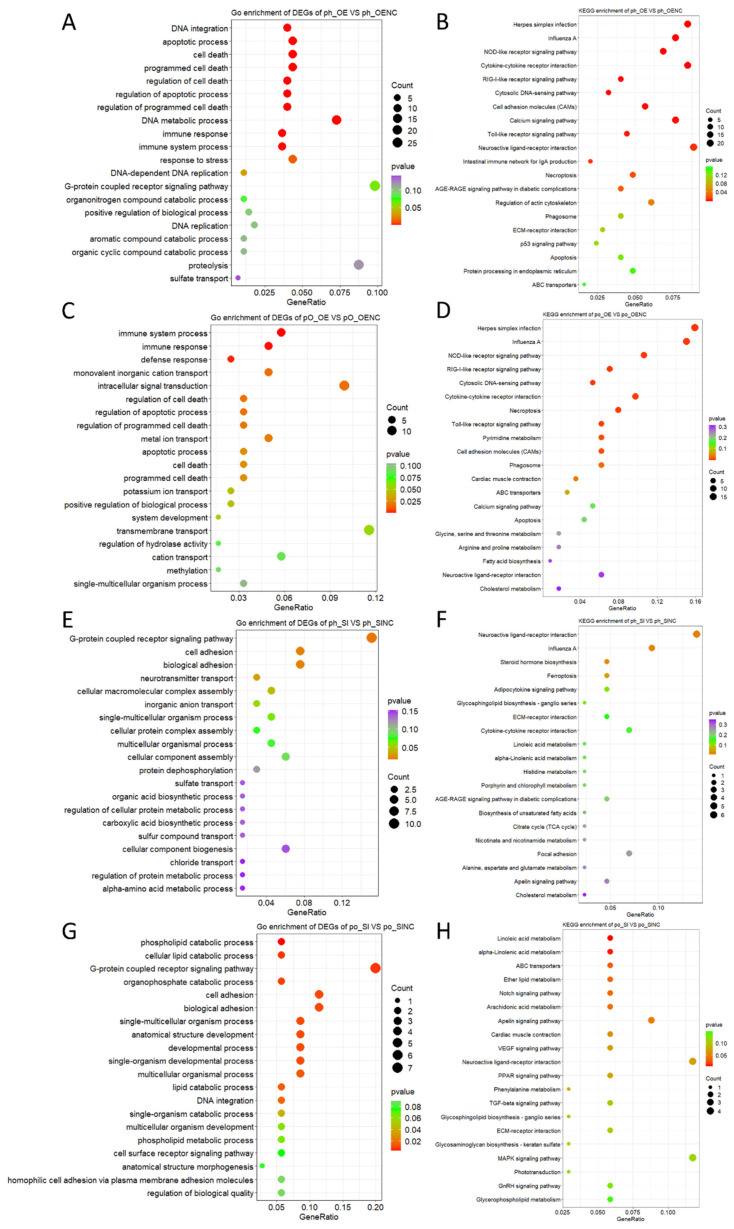
Functional enrichment analysis of the DEGs. (**A**,**B**) GO and KEGG analysis of DEGs in *FASN*-overexpressed phGCs, respectively. (**C**,**D**) GO and KEGG analysis of DEGs in *FASN*-overexpressed hGCs. (**E**,**F**) GO and KEGG analysis of DEGs in the *FASN*-interfered phGCs, respectively. (**G**,**H**) GO and KEGG analysis of DEGs in the *FASN*-interfered hGCs, respectively.

The biological process terms significantly enriched by DEGs in *FASN*-overexpressed phGCs and hGCs included the apoptotic process, cell death, immune response, immune system process, and so on (Figure 2A,C). It suggests that *FASN* overexpression might have an important impact on the immune and apoptosis of GCs. The KEGG pathways significantly enriched by these DEGs in the two categorized GCs included herpes simplex infection, influenza A, NOD-like receptor signaling pathway, Toll-like receptor signaling pathway, neuroactive ligand-receptor interaction, and so on (Figure 2B,D).

The biological process terms significantly enriched by DEGs in the *FASN*-interfered phGCs included the G-protein coupled receptor signaling pathway, cell adhesion, biological adhesion, and so on (Figure 2E). It suggests that *FASN* interference might have an important impact on the transmembrane signal transduction of phGCs. The KEGG pathways significantly enriched by these DEGs included neuroactive ligand-receptor interaction, influenza A, steroid hormone biosynthesis, and ferroptosis (Figure 2F).

The biological process terms significantly enriched by DEGs in the *FASN*-interfered hGCs included the phospholipid catabolic process, cellular lipid catabolic process, G-protein coupled receptor signaling pathway, and so on (Figure 2G). It suggests that *FASN* interference might have an important impact on the lipid metabolism and transmembrane signal transduction of hGCs. The KEGG pathways significantly enriched by these DEGs included linoleic acid metabolism, alpha-linolenic acid metabolism, and so on (Figure 2H).

### 2.5. Network Analysis for DEGs in FASN-Overexpressed phGCs and hGCs

**Figure 3:**
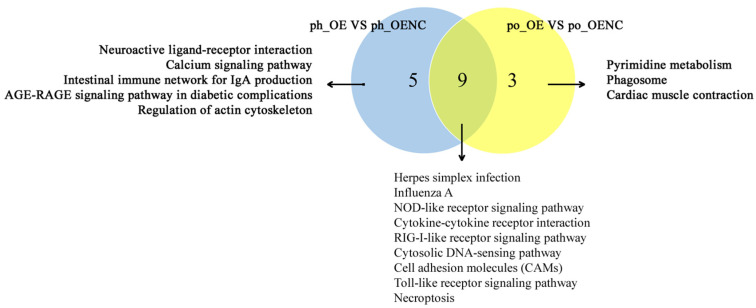
The intersection of significantly enriched pathways in the *FASN*-overexpressed phGCs and hGCs. ph_OE and po_OE are the abbreviations of the *FASN*-overexpressed phGCs and hGCs, respectively, while ph_OENC and po_OENC are the corresponding control groups, respectively. The number “9” indicate the significantly enriched pathways present both in the *FASN*-overexpressed phGCs and hGCs, while the number “5” and “3” indicate the significantly enriched pathways present only in the *FASN*-overexpressed phGCs and hGCs, respectively.

The common KEGG pathways enriched by DEGs in *FASN*-overexpressed phGCs and hGCs were screened including herpes simplex infection, influenza A, the NOD-like receptor signaling pathway, cytokine-cytokine receptor interaction, RIG-I-like receptor signaling pathway, cytosolic DNA-sensing pathway, cell adhesion molecules (CAMs), Toll-like receptor signaling pathway, and necroptosis (Figure 3). These nine pathways were all related to immune or necroptosis.

**Figure 4:**
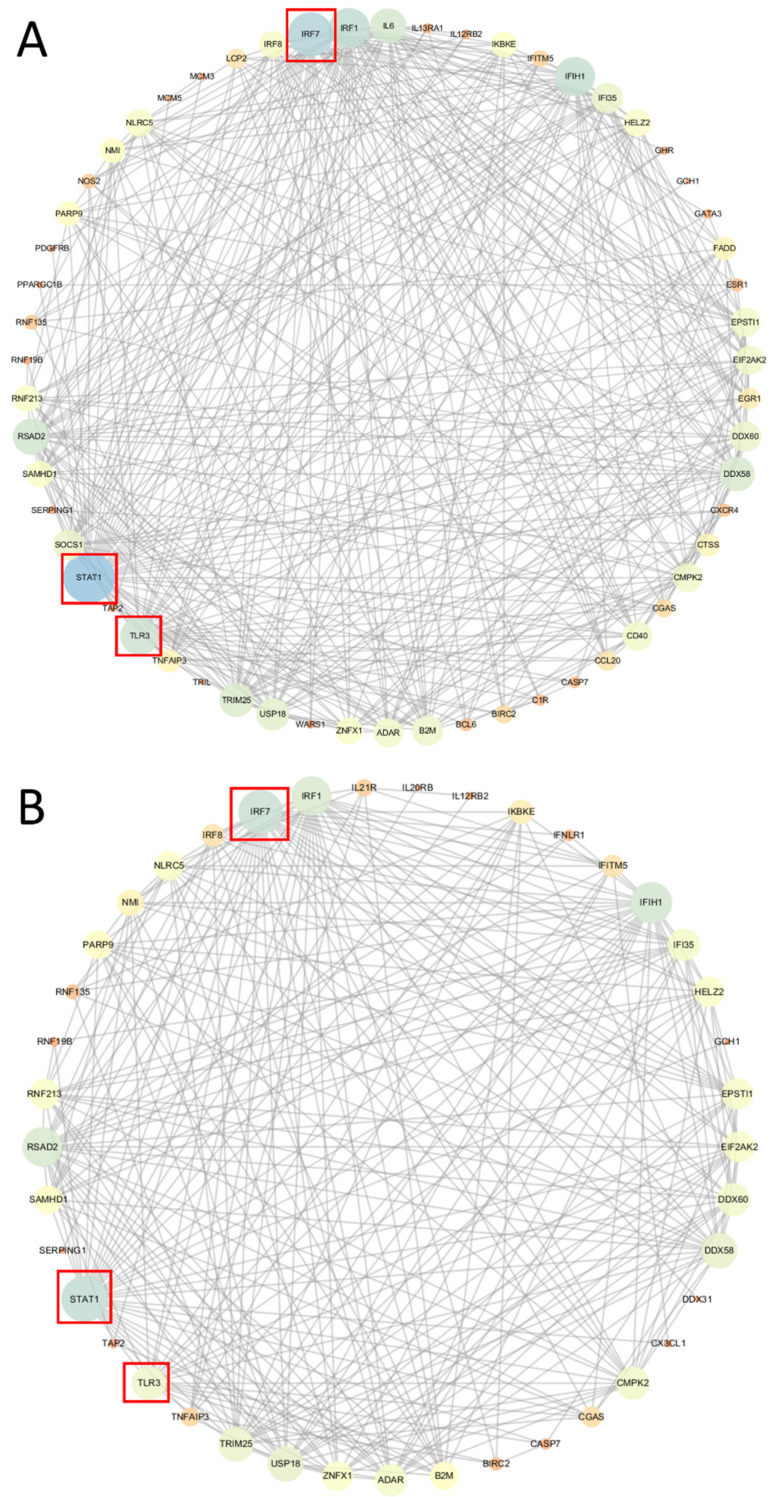
Network diagram of hub genes. (**A**,**B**) Network diagram of the top three hub genes in the *FASN*-overexpressed phGCs and hGCs, respectively. The nodes in the figure were proteins, and the connections were interactions. In the interaction network diagram, the size of the node was proportional to the degree of this node, where the greater the degree, the larger the node. The hub genes were circled in red.

To further explore the interaction among DEGs, the DEGs in these nine pathways were singled out and the interaction network was constructed using Cytoscape software (v 3.7.1, (National Institute of General Medical Sciences (NIGMS), Bethesda, Maryland, USA)). According to the score (ranked by degree method) of the nodes (Table S5), signal transducer and activator of transcription 1 (*STAT1*), interferon regulatory factor 7 (*IRF7*), and Toll-like receptors 3 (*TLR3*) were identified as the top three hub genes in the interaction networks of the *FASN*-overexpressed phGCs and hGCs. Then, we separated DEGs that interacted with *STAT1*, *IRF7*, and *TLR3* to draw the network diagram. In phGCs, the network showed that interactions mainly occurred among 16 immune-, seven cytokine-cytokine receptor interaction-, seven necroptosis-, and five apoptosis-related genes, respectively (Figure 4A). In hGCs, the network showed that interactions mainly occurred among 15 immune-, six necroptosis-, four apoptosis-, and five cytokine-cytokine receptor interaction-related genes, respectively (Figure 4B). However, the interaction networks of the *FASN*-interfered phGCs and hGCs were not constructed due to the small number of DEGs identified in the significantly enriched KEGG pathways.

### 2.6. Combined Metabolome and Transcriptome Analysis

**Figure 5:**
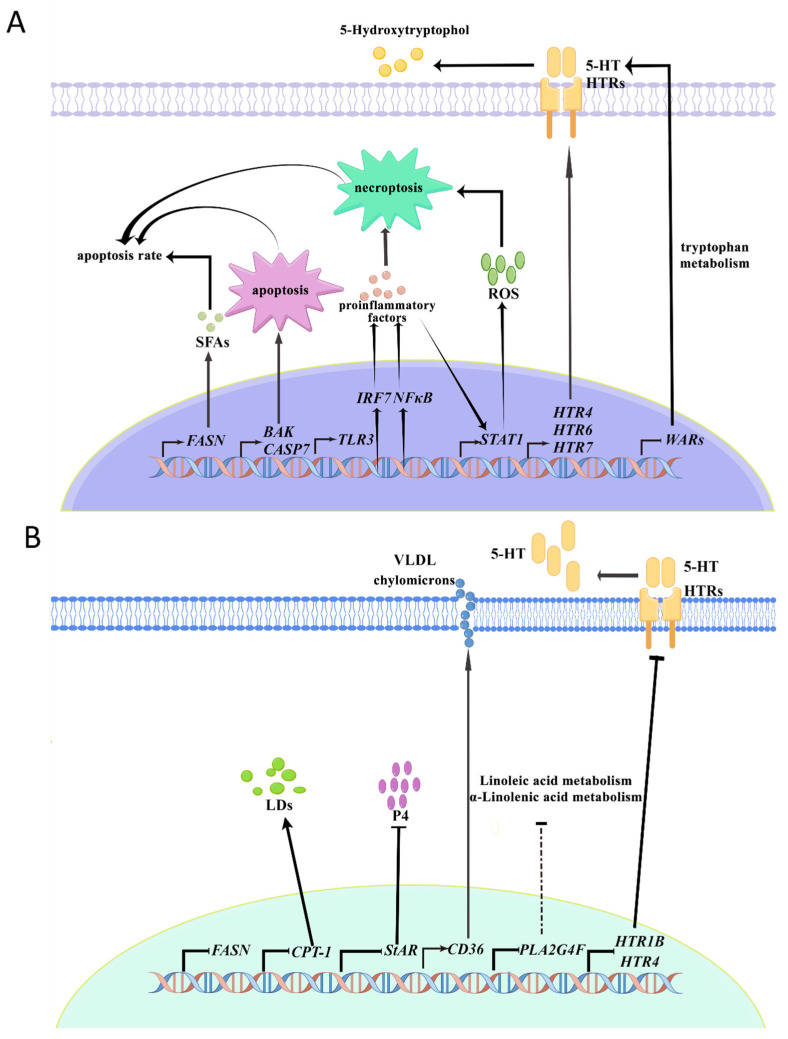
Effects of *FASN* on the function of *geese* GCs. (**A**) Effects of *FASN* overexpression on the function of phGCs. (**B**) Effects of *FASN* interference on the function of hGCs. This figure was drawn by Figdraw (www.figdraw.com (accessed on 15 April 2022)).

RNA-Seq analysis showed that *FASN* overexpression activated the neuroactive ligand-receptor interaction signal pathway in phGCs, upregulated the expression of *HTR4*, *HTR 6*, and *HTR7* (5-HT receptor 4, 6 and 7, the members of neuroactive ligand-receptor interaction signal pathway). In addition, *FASN* overexpression increased the content of 5-hydroxytryptophol (the metabolite of 5-HT), however, the content of 5-HT in the culture media of phGCs did not decrease (Figure 5A).

*FASN* interference also activated the neuroactive ligand-receptor interaction signal pathway in hGCs and downregulated the expression of *HTR1B* and *HTR4* (5-HT receptor 1B and 4, the members of neuroactive ligand-receptor interaction signal pathway). In addition, *FASN* interference increased the content of 5-HT in the culture media of hGCs (Figure 5B).

## 3. Discussion

In this study, we reported a comprehensive analysis of the transcriptome and metabolome of *FASN*-overexpressed GCs and *FASN*-interfered GCs for the identification of the mechanism of *FASN* regulating *geese* GC function.

Our results showed that the DEGs identified in the *FASN*-overexpressed phGCs and hGCs were both enriched in multiple immune related pathways such as the Toll-like receptor signaling pathway. *FASN* overexpression upregulated the expression of *TLR3* in the two categorized GCs. Endogenous molecules of nonmicrobial origin including SFAs were reported to modulate the activation of *TLRs*, which means that *TLRs* might be involved in sterile inflammation and immune response [7]. *TLRs* could mediate the expression of inflammatory cytokines such as interleukin-6 (*ILs*), interferons (*IFNs*), and tumor necrosis factors (*TNFs*) through the activation of transcription factors such as nuclear factor kappa β (*NF-kB*) and interferon regulatory factor (*IRFs*) [8,9]. Basically consistent with these above conclusions, our result showed that with the activation of *TLR3* induced by *FASN* overexpression, *IRF7* was upregulated in the two categorized GCs and *NF-kB2* was upregulated only in phGCs. It has been reported that exogenous SFAs were considered to be potential proinflammatory mediators and could induce the secretion of IL-6 through stimulating the Toll-like receptor signaling pathway [10]. Thus, it could be speculated that the excessive SFAs induced by *FASN* overexpression triggered the inflammatory response through activating *TLR3*. Then, *TLR3* subsequently enhanced the expression of the pro-inflammatory cytokines via the *TLR3*/*IRF7* pathway in the two categorized GCs, and only via the *TLR3*/*IRF7* signal pathways in hGCs. Our previous study revealed that the innate and acquired immunity in the *geese* ovary is closely related to reproductive performance [11]. There was evidence indicated that inflammatory cytokines participated in the regulation of GC function, for instance, *IL-6* was confirmed to be involved in the production of E_2_ and P_4_ in *human* GCs [12]. It could be inferred that this might also be one of the ways by which *FASN* regulates GCs function and indirectly affected the egg performance of *geese*.

We further identified the common top three hub genes (namely *STAT1*, *IRF7*, and *TLR3*) in the *FASN*-overexpressed phGCs and hGCs. These three genes were all upregulated by *FASN* overexpression and enriched into most immune- or necroptosis-related pathways. Necroptosis is the defense mechanism or escape route of cells in the face of infection or damage, which is inflammatory. After the activation of necroptosis, the exudation of the cell contents also triggers immune response. A previous study showed that the activation of *TLR3* could induce apoptosis in *mice* GCs [13]. *STAT1* is generally considered as a tumor suppressor, and the activation of *STAT1* has been confirmed to induce the apoptosis of *porcine* GCs [14]. In this study, our results suggest that the expression of *BAK* (*BCL-2* homologous antagonist/killer) and *caspase-7* were upregulated by *FASN* overexpression in both phGCs and hGCs. *BAK* can trigger apoptosis by inducing mitochondrial outer membrane permeability (MOMP) [15]. MOMP promotes the release of cytochrome C and other factors from the membrane gap to form apoptotic bodies, and then activates the *caspase* cascade, which irreversibly leads to cell death [16,17]. This was basically consistent with the conclusion of the research in *mice* and *porcine* GCs, as above-mentioned.

After *FASN* interference, two transcriptional subtypes of DEGs, namely, *aromatase* and *3βHSD*, were enriched into the steroid hormone biosynthesis pathway in phGCs. These two enzymes are responsible for E_2_ [18] and P_4_ synthesis [19]. Therefore, it could be speculated that *FASN* interference might inhibit the steroidogenesis of phGCs. Several fatty acid metabolic pathways were enriched by DEGs in *FASN*-interfered hGCs including linoleic acid metabolism, alpha-linolenic acid metabolism, and so on. *PLA2G4F* (*PLA2*, group IVF) was one of the DEGs enriched into these above pathways, and its expression was downregulated by *FASN* interference. The activation of *PLA2G4F* could induce the release of polyunsaturated fatty acids from cell membrane phospholipids [20]. However, this process in hGCs was inhibited by *FASN* interference. It was speculated that this may be an adjustment made by hGCs in order to minimize the hydrolysis of membrane phospholipids and give priority to maintaining the structure and function of cell membranes when DNL is inhibited.

Next, we performed a combined analysis for the results of the transcriptome and metabolome, and confirmed that the neuroactive ligand-receptor interaction pathway was crucial for *FASN* to regulate the function of *geese* phGCs and hGCs. Several transcriptome studies have confirmed the important role of the neuroactive ligand-receptor interaction pathway in the regulation of reproductive activities such as studies in *hen* [21], *pig* [22], and *goat* [23]. In addition, our previous study suggested that the neuroactive ligand-receptor interaction pathway, especially the *HTRs* family, was pivotal for *geese* egg performance [11]. In this study, the results showed that *FASN* interference downregulated the expression of *HTR1B* and *HTR4* in hGCs, resulting in decreased metabolic demand for 5-HT in hGCs. Meanwhile, we detected that the content of 5-HT in the culture medium of *FASN*-interfered hGCs increased. *FASN* overexpression upregulated the expression of *HTR4*, *HTR6,* and *HTR7* in phGCs, resulting in an increased metabolic demand for 5-HT in phGCs. Meanwhile, we detected that the content of 5-hydroxytryptophol (a metabolite of 5-HT), in the culture medium of *FASN*-overexpressed phGCs increased. Interestingly, however, with the enhanced metabolism of 5-HT in *FASN*-overexpressed phGCs, no decrease in 5-HT content was detected in the culture medium. Combined with the transcriptome results, this might be caused by the change in tryptophan metabolism in the phGCs. 5-HT is derived from tryptophan, and brain-derived 5-HT accounts for about 5% of the total 5-HT in the body, while the remaining 95% of 5-HT is produced by peripheral organs [24]. The excessive free fatty acids (FFAs) in plasma have been reported to stimulate platelets to secrete more 5-HT, resulting in an increase in the concentration of 5-HT in plasma [25]. Our transcriptome results showed that in phGCs, the expression of *WAR* (tryptophan tRNA synthetase) was downregulated by *FASN* overexpression, which meant that the amount of tryptophan involved in protein synthesis decreased. It could be speculated that this part of tryptophan may be used for 5-HT synthesis and continuously secreted into the culture medium to make up for the consumption of 5-HT. This revealed that *FASN* overexpression activated the tryptophan metabolic pathway of phGCs, inducing phGCs to improve the ability to metabolize tryptophan to 5-HT, so as to adapt to the change of 5-HT demand caused by *FASN* overexpression. However, more in-depth researches are needed to further understand the role of 5-HT and *HTRs* in the GC function and egg performance of *geese*.

## 4. Materials and Methods

### 4.1. Experimental Animals

The maternal line of Tianfu meat *geese* (35–40 weeks of age) laying in regular sequences of at least 2–3 eggs were selected for this study. These *geese* were kept under the same conditions of light and temperature and were allowed ad libitum to feed and water at the Waterfowl Breeding Experimental Farm at Sichuan Agricultural University (Sichuan, China). Individual laying cycles were monitored and recorded, and the healthy *geese* were euthanized by cervical dislocation 6–8 h ahead of oviposition. All experiments were conducted according to the institutional ethical guidelines for animal experiments of the National Defense Medical College. These experiments were approved by the Sichuan Agricultural University Animal Welfare Committee (approval number: 20190035, date of approval 12 March 2019).

### 4.2. GC Culture and Transfection

The granulosa layers separated from the pre-hierarchical follicles (6–10 mm) and hierarchical follicles (F4-F1) were washed with PBS (Solar bio, Beijing, China), respectively. GCs were isolated by 0.1% collagenase Ⅱ (Sigma, Aldrich, Burlington, MA, USA) digestion, then counted and cultured as previously described [26]. At 70~80% confluence, GCs were transfected with *pEGFP-N1-FASN* (2 μg/mL), *pEGFP-N1* (2 μg/mL, control) and siRNA-*FASN* (90 pmol/mL), siRNA-NC (90 pmol/mL, control) individually using lipofectamine 3000 (Invitrogen, Carlsbad, CA, USA) according to the manufacturer’s instructions, and assayed at 48 h post-transfection [5].

### 4.3. Metabolomics Detection

We divided 48 samples into four groups, and each group was further divided into two groups (experimental group and control group) (Table 4). A total of 100 μL of each sample was added to a 1.5 mL Eppendorf tube and then resuspended with prechilled 80% methanol and 0.1% formic acid by well vortexing. The samples were incubated on ice for 5 min and then centrifuged at 15,000 rpm, 4 °C for 5 min. Some of the supernatant was diluted to a final concentration containing 60% methanol by LC-MS grade water. The samples were subsequently transferred to a fresh Eppendorf tube with a 0.22 μm filter and then centrifuged at 15,000× *g*, 4 °C for 10 min. Finally, the filtrate was injected into the LC-MS/MS system analysis. In addition, the QC sample was prepared by mixing equal aliquots of all samples to generate a pooled sample.

LC-MS/MS analyses were performed using a Vanquish UHPLC system (Thermo Fisher, Waltham, MA, USA) coupled with an Orbitrap Q Exactive HF-X mass spectrometer (Thermo Fisher, Waltham, MA, USA). Samples were injected onto a Hyperil Gold column (100 × 2.1 mm, 1.9 μm) using a 16-min linear gradient at a flow rate of 0.2 mL/min. The eluents for the positive polarity mode were eluent A (0.1% FA in water) and eluent B (methanol). The eluents for the negative polarity mode were eluent A (5 mM ammonium acetate, pH 9.0) and eluent B (methanol). The solvent gradient was set as follows: 2% B, 1.5 min; 2–100% B, 12.0 min; 100% B, 14.0 min; 100–2% B, 14.1 min; 2% B, 16 min. Q Exactive HF-X mass spectrometer was operated in positive/negative polarity mode with a spray voltage of 3.2 kV, capillary temperature of 320 °C, sheath gas flow rate of 35 arb, and aux gas flow rate of 10 arb.

### 4.4. Metabolites Identification

The raw data files generated by UHPLC-MS/MS were processed using the Compound Discoverer 3.0 (CD 3.0, Thermo Fisher, Waltham, MA, USA) to perform peak alignment, peak picking, and quantitation for each metabolite. The main parameters were set as follows: retention time tolerance, 0.2 min; actual mass tolerance, 5 ppm; signal intensity tolerance, 30%; signal/noise ratio, 3; and minimum intensity, 100,000. Afterward, the peak intensities were normalized to the total spectral intensity. The normalized data were used to predict the molecular formula based on additive ions, molecular ion peaks, and fragment ions. Then, the peaks were matched with the mzCloud (https://www.mzcloud.org/ (accessed on 15 September 2019), HighChem LLC, Kashiwa, Chiba, Japan) and ChemSpider (http://www.chemspider.com/ (accessed on 15 September 2019), Royal Society of Chemistry, Science Park, Milton Road, Cambridge, UK) database to obtain accurate qualitative and relative quantitative results.

### 4.5. Differential Metabolite Analysis

Projection to partial least-squares-discriminant analysis (PLS-DA) was further conducted to obtain a high level of group separation and to better understand the variables responsible for group classification. To guard against overfitting, the default 7-fold cross-validation was applied and permutation tests with 200 iterations were performed to further validate the supervised model. Based on the combination of a statistically significant threshold of variable influence on the projection values (VIP > 1) calculated from the PLA-DA model and the *p*-value (*p* < 0.05), the differential metabolites were selected.

### 4.6. RNA-Seq and Functional Analysis of the DEGs

We divided 32 samples into four groups, and each group was further divided into two groups (experimental group and control group) (Table 4), and the total RNA was extracted using a RNeasy Mini Kit (Qiagen, Germany) at Novogene (Tianjin, China). The integrity of total RNA was detected with the RNA Nano 6000 Assay Kit of the Bioanalyzer 2100 system (Agilent Technologies, Santa Clara, CA, USA).

Then, the cDNA libraries were constructed using the NEBNext^®^ UltraTM RNA Library Prep Kit for Illumina^®^ (NEB, Ipswich, MA, USA) following the manufacturer’s recommendations and sequenced on an Illumina Hiseq platform. The adaptor reads, poly-N contained reads (N% >10%), and low-quality reads were removed, and the clean reads were mapped onto *geese* reference genome (anscyg_prjna183603_v1.0) by Hisat2 (v2.0.5). The read numbers mapped to each gene were counted using featureCounts v1.5.0-p3, then the fragments per kilobases per million mapped reads (FPKM) of each gene was calculated based on the length of the gene and reads count mapped to this gene. Comparisons of FPKM in the four groups were performed using the DESeq2 R package (1.16.1), and the genes with a fold change (FC) more than 1.5 and *p*-value less than 0.05 were defined as DEGs. Next, the DEGs were subjected to Gene Ontology (GO) and Kyoto Encyclopedia of Genes and Genomes (KEGG) analysis by the clusterProfiler R package, and the GO terms and KEGG pathways with a *p*-value less than 0.05 were considered significantly enriched by DEGs.

### 4.7. qRT-PCR for Validation of the DEGs

Twelve genes (*TLR3*, *EIF2AK2*, *FADD*, *IKBKE*, *IRF7*, *BID*, *BIRC2*, *MAP3K8*, *STAT1*, *TNFAIP3*, *AGT*, *HMOX1*) were selected for validation by qRT-PCR using 2 × SYBR Premix Ex Taq II (TaKaRa, Dalian, China). The reaction solutions were prepared in a total volume of 12.5 μL containing 1 μL cDNA, 6.25 μL of 2 × SYBR Premix Ex Taq, 4.25 μL of ddH_2_O, and 0.5 μL of each gene-specific primer (10 μM). For each sample, the analysis was conducted in triplicate and normalized to *β-actin* by the 2^−ΔΔCt^ method [27]. The control was set as one. The primers for qRT-PCR are summarized in Table 5.

## 5. Conclusions

We showed that the DEGs in the *FASN*-overexpressed phGCs and hGCs were mostly enriched into immunity- and necroptosis-related pathways, especially the Toll-like receptor signal pathway. *FASN* overexpression could promote proinflammatory factor expression by activating the *TLR3*/*IRF7* and *TLR3*/*NF-κB* signal pathways in phGCs, and only by activating the *TLR3*/*IRF7* signal pathways in hGCs. These proinflammatory factors could induce the necroptosis of *geese* GCs by activating the *JAK*/*STAT1* signaling pathway. In addition, *FASN* overexpression also induced the apoptosis of *geese* GCs by activating the *BAK*/*caspase-7* signaling pathway. The combined analysis of the metabolome and transcriptome revealed that *FASN* affected the demand of GCs for 5-HT by activating the neuroactive ligand-receptor interaction pathway in two categorized GCs and only altering the metabolic pathway of tryptophan in phGCs, and ultimately participated in regulating the physiological function of *geese* GCs and follicle development. Based on these findings, the obtained results provide a theoretical basis for further analyzing the mechanism of lipid metabolism involved in the regulation of GC function and follicular development in *geese*. However, further studies are needed to gain a deeper understanding of the roles of 5-HT and *HTRs* in the GC function and egg performance of *geese*.

## Figures and Tables

**Table 1:** The number of differential metabolites.

Compared Samples	Num. of Total Sig.	Num. of Sig. Up	Num. of Sig. Down
ph_OE vs. ph_OENC	63	59	4
po_OE vs. po_OENC	54	33	21
ph_SI vs. ph_SINC	36	19	17
po_SI vs. po_SINC	35	17	18

Note: ph_OE and po_OE are the abbreviations of *FASN*-overexpressed phGCs and hGCs, respectively, while ph_OENC and po_OENC are the corresponding control groups, respectively. ph_SI and po_SI are the abbreviations of *FASN*-interfered phGCs and hGCs, respectively, while ph_SINC and po_SINC are the corresponding control groups, respectively.

**Table 2:** Significantly enriched KEGG pathway by differential metabolites.

Group	Map ID	Map Title	*p*-Value
ph_OE vs. ph_OENC	map00240	Pyrimidine metabolism	0.019931862
	map00520	Amino sugar and nucleotide sugar metabolism	0.042016807
	map00480	Glutathione metabolism	0.031746032
ph_SI vs. ph_SINC	map00780	Biotin metabolism	0.015873016
po_SI vs. po_SINC	map04726	Serotonergic synapse	0.001424299
	map04080	Neuroactive ligand-receptor interaction	0.001914757
	map04540	Gap junction	0.004199855
	map04742	Taste transduction	0.004199855
	map04750	Inflammatory mediator regulation of TRP channels	0.004199855
	map04024	cAMP signaling pathway	0.008255516
	map04721	Synaptic vesicle cycle	0.008255516
	map03320	*PPAR* signaling pathway	0.042016807
	map04261	Adrenergic signaling in cardiomyocytes	0.042016807
	map04270	Vascular smooth muscle contraction	0.042016807
	map04924	Renin secretion	0.042016807
	map04970	Salivary secretion	0.042016807

Note: ph_OE and po_OE are the abbreviations of the *FASN*-overexpressed phGCs and hGCs, respectively, while ph_OENC and po_OENC are the corresponding control groups, respectively. ph_SI and po_SI are the abbreviations of the *FASN*-interfered phGCs and hGCs, respectively, and ph_SINC and po_SINC are the corresponding control groups, respectively.

**Table 3:** The number of DEGs.

Group	Num. of DEG	Num. of Sig. Up	Num. of Sig. Down
ph_OE vs. ph_OENC	1099	724	375
po_OE vs. po_OENC	591	373	128
ph_SI vs. ph_SINC	224	102	122
po_SI vs. po_SINC	161	70	91

Note: ph_OE and po_OE are the abbreviations of the *FASN*-overexpressed phGCs and hGCs, respectively, and ph_OENC and po_OENC are the corresponding control groups, respectively. ph_SI and po_SI are the abbreviations of the *FASN*-interfered phGCs and hGCs, respectively, and ph_SINC and po_SINC are the corresponding control groups, respectively.

**Table 4:** The grouping situation in this study.

Group	Experimental Group	Control Group
ph_OE vs. ph_OENC	phGCs transfected with *pEGFP-N1-FASN*	phGCs transfected with *pEGFP-N1*
po_OE vs. po_OENC	hGCs transfected with *pEGFP-N1-FASN*	hGCs transfected with *pEGFP-N1*
ph_SI vs. ph_SINC	phGCs transfected with *siRNA-FASN*	phGCs transfected with *siRNA-NC*
po_SI vs. po_SINC	hGCs transfected with *siRNA-FASN*	hGCs transfected with *siRNA-NC*

Note: ph_OE and po_OE are the abbreviations of the *FASN*-overexpressed phGCs and hGCs, respectively, and ph_OENC and po_OENC are the corresponding control groups, respectively. ph_SI and po_SI are the abbreviations of the *FASN*-interfered phGCs and hGCs, respectively, and ph_SINC and po_SINC are the corresponding control groups, respectively.

**Table 5:** Primers for qRT-PCR.

Genes	AccessionNumber	Primer (5′-3′)	Tm (°C)	Size (bp)
*TLR3*	KC292270.1	F:CCCCCAGACTGAAGGAGGTAR:TGACATTGCTTTCCGACCGA	63	129
*EIF2AK2*	XM_013170695.1	F:GACCTCACCATGTGGACCAGR:CTTCTTTTGCAGCGGCTTGT	63	227
*FADD*	XM_013194898.1	F:ACCATGGACCCGTTTCTGACR:AGGAAGTTGAAGAGCTCCGC	63	149
*IKBKE*	XM_013189616.1	F:GGAGCATCGAGTGGAGCTAC R:TCGAACCCCCAGCATTTCTC	65	124
*IRF7*	XM_013174398.1	F:CAGCAGCGACATCGAGATCTR:TCGCTGTTCTTGGAGTGGTC	63	156
*BID*	XM_013172977.1	F:CGAGAGAAGGCCATGTTGGTR:GCTGGAGATCTCTCACTCGC	63	162
*BIRC2*	XM_013178135.1	F:CTCTGGTACCTGGAGCTCCT R:AGAATGGACTCACTGCTGGC	63	179
*MAP3K8*	XM_013191750.1	F:ATTCCTCGGGGAGCTTTTGGR:GATGGATCGTCTCGTCCCAC	63	184
*STAT1*	XM_013187488.1	F:GGATCTCAAAACGAGCCCCAR:ATGTAGCCGTTTCCCTTGGG	63	245
*TNFAIP3*	XM_013175520.1	F:GACAGCCCGAAAGAACAGGAR:CACAGCTCCTCACTTCTGCA	63	107
*AGT*	XM_048045900.1	F:AAACAGGGATGAGGAGCTGCR:TGGCCTCCACAAAGGTGTTT	63	182
*HMOX1*	XM_013181078.2	F:GGAGGAGGCCAAGAAAGCATR:GCCTTTGACTGCACAAGGTG	63	105
β-actin *	M26111.1	F:CAACGAGCGGTTCAGGTGTR:TGGAGTTGAAGGTGGTCTCG	60	170

Note: F: sense primers; R: antisense primers. * House-keeping gene for data normalization.

## Data Availability

The data in this study can be shared upon reasonable request from the corresponding author.

## Supplementary Materials

The following supporting information can be downloaded at: https://www.mdpi.com/article/10.3390/ijms232314717/s1.
