# Supplementary material for: Integrated Transcriptome and Metabolome Analysis Reveals the Regulatory Mechanisms of FASN in Geese Granulosa Cells
Source: Int J Mol Sci. 2022 Nov 25;23(23):14717. doi: 10.3390/ijms232314717 (PMC9736573; doi:10.3390/ijms232314717)
Supplement: Supplementary file 1 [file ijms-23-14717-s001.zip › ijms-2032364-supplementary Table S1.pdf]

Table S1. Differential metabolites

group	ID	metabolites	log_2FC	pvalue	VIP	Up.Down
ph_OE *vs* ph_OENC	Com_755_pos	Cytosine	0.297663955	4.39E-05	1.350759806	up
	Com_7011_pos	Jasmonal	0.42022651	0.001047489	1.916662081	up
	Com_2036_pos	(E)-2-methylgeranyl diphosphate	0.380395684	0.003858023	1.730877695	up
	Com_8114_pos	TH9185000	0.526872586	0.004238472	2.356031091	up
	Com_1183_pos	MFCD00010043	0.311429118	0.004913762	1.38660409	up
	Com_8612_pos	Furaspor	0.374791719	0.005193462	1.738995005	up
	Com_6923_pos	LV1850000	0.270943849	0.006696469	1.251399308	up
	Com_2956_pos	zidometacin	0.534544628	0.006847604	2.484419988	up
	Com_8650_pos	Propoxur	0.378732109	0.007210016	1.719508174	up
	Com_2145_pos	gitogenin	0.320397085	0.007281778	1.433459589	up
	Com_7883_pos	N-Acetylneuraminic acid	0.464952488	0.009278344	2.020570456	up
	Com_8557_pos	AMPA	0.695432358	0.009388508	3.231450011	up
	Com_5039_pos	N~6~,N~6~-Dimethyllysine	0.315237082	0.010055109	1.452629465	up
	Com_9077_pos	2,4-Diphenyl-1-butene	0.738910189	0.010645294	3.349374484	up
	Com_7537_pos	(+/-)-2-Hydroxyglutaric acid	0.422875498	0.012542459	1.890061612	up
	Com_2005_pos	Toxopyrimidine	0.391416515	0.012917609	1.757249468	up
	Com_7907_pos	hymecromone	0.356258766	0.013292938	1.580228695	up
	Com_7442_pos	ibufenac	0.325793075	0.016070359	1.562025297	up
	Com_2277_pos	1-icosanoyl-sn-glycero-3-phosphocholine	0.450171902	0.017824941	2.157132579	up
	Com_3887_pos	furilazole	0.271797581	0.018341147	1.195732061	up
	Com_2257_pos	3-Acetyl-2,5-dimethylfuran	0.466481687	0.018604541	2.063046803	up

Continued table S1

	Com_2431_pos	2-n-Butyl-4-ethyl-5-methyloxazole	0.370069929	0.019239102	1.698042666	up
	Com_2805_pos	4-Methyl-5-thiazoleethanol	0.429718409	0.020141928	1.887539219	up
	Com_4733_pos	S(8)-(2-methylpropanoyl)dihydrolipoamide	0.327349321	0.020510962	1.515237074	up
	Com_3870_pos	SKF-97,541	0.563130153	0.020556444	2.684624154	up
	Com_4249_pos	lymecycline	0.389579594	0.02078911	1.799913722	up
	Com_743_pos	2-Methylthiazolidine	0.298989381	0.021970853	1.308336432	up
	Com_2249_pos	Ethosuximide	0.270057826	0.024240884	1.208484181	up
	Com_4370_pos	Niraparib	0.406343648	0.024570496	1.861758332	up
	Com_3858_pos	Arachidonic acid ethyl ester	0.809457411	0.026885951	3.3183009	up
	Com_1291_pos	Piperazine	0.426039283	0.028361848	2.107467866	up
	Com_8358_pos	Hydantoin-5-propionic acid	0.411063923	0.029782512	1.919029041	up
	Com_8698_pos	fenbuconazole	0.450367884	0.03244887	2.226897692	up
	Com_6370_pos	ET9020000	0.276461066	0.032664874	1.244262197	up
	Com_4893_pos	5-Indolol	0.300240524	0.033749821	1.292028521	up
	Com_6099_pos	5-Hydroxytryptophol	0.478457563	0.033847366	2.425230799	up
	Com_4931_pos	Prednisolone farnesylate	0.404455208	0.035098481	1.893966725	up
	Com_2203_pos	KL2880000	0.27237095	0.036695223	1.266618545	up
	Com_2480_pos	Arabinosylhypoxanthine	0.30949838	0.037414651	1.420578338	up
	Com_2072_pos	LQ1825000	0.286000712	0.03904336	1.349547939	up
	Com_3317_pos	NI3400000	0.405654239	0.039371614	2.026852939	up
	Com_5650_pos	palmatine	0.325631567	0.03962823	1.536056578	up
	Com_1692_pos	Cytidine	0.554742663	0.04043151	2.633654671	up
	Com_8237_pos	Bethanechol	0.318558014	0.041112358	1.384437924	up

Continued table S1

	Com_6704_pos	1,4-Dimethylimidazole	0.463579493	0.041141832	2.119632064	up
	Com_6521_pos	malonamic acid	0.358944686	0.042198904	1.757864735	up
	Com_8517_pos	Tyramine	0.323849624	0.04511859	1.581075957	up
	Com_690_pos	Tris(2-chloroethyl) phosphate	-0.941376329	0.046448995	3.724370761	down
	Com_2767_pos	13-Hydroxykaur-16-en-18-oic acid	0.364173161	0.046896737	1.582535826	up
	Com_7064_pos	Mesalazine	0.349264313	0.049085123	1.584954386	up
	Com_9140_pos	N(4)-phosphoagmatine	0.389180398	0.049517498	1.867111699	up
	Com_2638_pos	LysoPC(P-18:0)	0.346296193	0.049775508	1.479405079	up
	Com_5076_neg	Callichiline	0.807448241	0.003550014	1.976264883	up
	Com_522_neg	γ-Linolenic acid	-0.533447047	0.009339822	1.304585086	down
	Com_1488_neg	dihydroconiferyl alcohol glucoside	0.774785573	0.014334123	1.726067184	up
	Com_4429_neg	Scopoletin	0.420639497	0.019047485	1.051161355	up
	Com_969_neg	butyrin	1.438489469	0.020631939	5.176895868	up
	Com_2367_neg	Glutathionylspermine	0.836271579	0.031273378	2.21870486	up
	Com_5055_neg	Guvacine	0.437116578	0.035208617	1.048989328	up
	Com_1800_neg	Losartan	0.938843245	0.035298192	2.638795043	up
	Com_436_neg	Dimethylsulfoniopropionate	-1.453632854	0.035327217	5.299240027	down
	Com_4428_neg	15S-hydroxyeicosatrienoic acid	0.488142989	0.038754891	1.109386174	up
	Com_271_neg	Myristyl sulfate	-0.482724082	0.048481444	1.061305025	down
ph_SI *vs* ph_SINC	Com_3575_pos	3, 5-Tetradecadiencarnitine	0.47300876	0.000489502	2.302043837	up
	Com_276_pos	Olodaterol	-1.476811663	0.001609942	7.13616549	down
	Com_2145_pos	gitogenin	0.267845896	0.002626149	1.301248736	up
	Com_2680_pos	MFCD00058970	-1.298766553	0.003022076	5.975026815	down

Continued table S1

Com_3466_pos	rutaevin	-1.34352687	0.003601081	6.30594935	down
Com_850_pos	Tamsulosin	-1.487385921	0.003787679	7.067291419	down
Com_9101_pos	phenicarbazide	0.417332261	0.005736409	1.993731779	up
Com_4574_pos	Sirodesmin G	-1.382977016	0.007199749	6.602679189	down
Com_1772_pos	2-Arachidonoyl glycerol	0.43231524	0.009998629	2.134983731	up
Com_4869_pos	LL8225000	0.361121573	0.014238651	1.767850506	up
Com_3585_pos	O-oleoylcarnitine	0.339564633	0.016613583	1.616765024	up
Com_1291_pos	Piperazine	0.421319443	0.019801414	1.975499764	up
Com_5687_pos	TIRON FREE ACID	0.333803247	0.019889049	1.636892538	up
Com_3440_pos	Hecogenin	0.31645938	0.021828446	1.498654222	up
Com_1446_pos	2-Amino-1,3,4-octadecanetriol	1.405834105	0.027746124	6.177185869	up
Com_390_pos	Luvangetin	0.263078573	0.029080519	1.329341068	up
Com_2073_pos	2-(14,15-Epoxyeicosatrienoyl) glycerol	0.396381251	0.032158228	1.964818717	up
Com_5735_pos	Methional	0.366265448	0.03330703	1.705138519	up
Com_5722_pos	Chemotactic peptide	0.289271911	0.037742741	1.435721888	up
Com_682_pos	Isoquinoline	0.268416648	0.044943184	1.359207702	up
Com_547_neg	Olmesartan	-1.460534868	0.00084627	3.809067817	down
Com_1800_neg	Losartan	-1.926535936	0.001776861	4.745502981	down
Com_747_neg	elfazepam	-0.641043459	0.002556013	1.664667379	down
Com_2493_neg	benthiocarb	-2.918363532	0.008278792	6.278453211	down
Com_3067_neg	(2'S)-Deoxymyxol 2'-alpha-L-fucoside	0.792834971	0.009086485	2.342574339	up
Com_2301_neg	Trichotomine	-1.965268332	0.014296973	4.141238647	down

Continued table S1

	Com_642_neg	Suvorexant	-1.380729899	0.015758055	3.755446864	down
	Com_4030_neg	ethaverine	-1.230817209	0.016551102	2.925983891	down
	Com_1807_neg	Octacosyl (2E)-3-(3-hydroxy-4-methoxyphenyl)acrylate	0.499285116	0.027254356	1.307674299	up
	Com_2358_neg	Nostocyclopeptide A3	0.640278113	0.029593575	1.722035106	up
	Com_1143_neg	Biocytin	-1.50689019	0.030160477	3.302288535	down
	Com_2984_neg	Nigakinone	-1.990336848	0.036310172	3.848583458	down
	Com_5152_neg	Chlorohyssopifolin A	0.45934822	0.039325184	1.207096706	up
	Com_187_neg	4-Dodecylbenzenesulfonic acid	-0.488252407	0.041457367	1.166561839	down
	Com_553_neg	Lisuride	-0.577404079	0.042915892	1.800623202	down
	Com_1378_neg	δ-Ribono-1,4-lactone	-0.362342316	0.048582431	1.072895411	down
po_OE *vs* po_OENC	Com_2005_pos	Toxopyrimidine	0.432284709	0.001644342	2.106811944	up
	Com_1789_pos	Stearoylethanolamide	0.41782754	0.001822953	2.067567581	up
	Com_3590_pos	Azathioprine	0.328496177	0.002335124	1.617904506	up
	Com_1772_pos	2-Arachidonoyl glycerol	0.464792992	0.002728713	2.276653687	up
	Com_2259_pos	2-(4-Methylpiperazino)-N-(2-phenoxyphenyl)acetamide	0.448188598	0.004506092	2.250741571	up
	Com_2145_pos	gitogenin	-0.326489384	0.005167692	1.607417456	down
	Com_7454_pos	(+)-Alantolactone	0.365585956	0.007189165	1.734331562	up
	Com_787_pos	6-Methoxyquinoline	-0.360206846	0.007656582	1.692470812	down
	Com_6537_pos	2,3,4,5-tetrahydrodipicolinic acid	-0.457735956	0.016642174	2.363209235	down
	Com_8444_pos	Cyanopyrazine	-0.282352736	0.017350764	1.38208359	down
	Com_4574_pos	Sirodesmin G	-0.861687889	0.019416829	4.23703016	down

Continued table S1

Com_8650_pos	Propoxur	-0.441030128	0.020471407	2.078758706	down
Com_1165_pos	tolimidone	0.299834904	0.02243333	1.443492438	up
Com_303_pos	Diisobutylphthalate	-0.397595274	0.023719132	2.034998279	down
Com_5265_pos	Phytosphingosine	-0.317161669	0.025003673	1.534976294	down
Com_6954_pos	N-Acetyl-L-aspartic acid	-0.397749828	0.03007235	1.961264274	down
Com_531_pos	laurilsulfate	0.27057734	0.030702648	1.322601753	up
Com_1622_pos	(1S,2R,5S)-2-Isopropyl-5-methylcyclohexyl 3-oxobutanoate	-0.508139112	0.031413954	2.408354923	down
Com_6378_pos	Stiripentol	0.264724358	0.0332829	1.360948723	up
Com_8519_pos	4-Nitrosobiphenyl	-0.336522735	0.037970987	1.697653527	down
Com_3968_pos	NECA	2.478175312	0.038483478	9.069288798	up
Com_1521_pos	9-(3-Methyl-5-pentyl-2-furyl)nonanoic acid	-0.955735558	0.039938454	3.923869052	down
Com_1567_pos	p-Chloroacetophenone	0.728147052	0.040620658	3.508976105	up
Com_5612_pos	Leukotriene E4	-0.4368671	0.044630117	1.944053532	down
Com_7344_pos	DK3970000	0.301009201	0.045320602	1.426797128	up
Com_7500_pos	UX9640000	-0.40099104	0.045972536	1.825569885	down
Com_8326_pos	Menadiol	-0.330091202	0.048996052	1.528546372	down
Com_6747_pos	protocatechuic acid	-0.300605463	0.049504796	1.514343951	down
Com_2789_neg	4,4'-DBP	-0.305562786	0.004284757	1.098459233	down
Com_923_neg	2,3-Dimethylquinoxaline	-0.289979871	0.005479496	1.072796503	down
Com_2038_neg	2,2'-Bipyridine	-0.355182844	0.007204217	1.324332836	down
Com_3390_neg	3-Hydroxytetradecanedioic acid	-0.278782599	0.010522744	1.003727312	down
Com_2984_neg	Nigakinone	-0.972342437	0.016071971	4.242784818	down

Continued table S1

	Com_2493_neg	benthiocarb	-1.282733475	0.017898814	6.560650571	down
	Com_473_neg	1-Oxo-1,2,4-butanetricarboxylic acid	-0.346682981	0.018165674	1.335024559	down
	Com_2309_neg	Kukoamine A	0.759981134	0.022389639	2.602044502	up
	Com_2206_neg	CAPSO	0.8971306	0.023010188	3.019952062	up
	Com_1420_neg	L-adenosylselenohomocysteine	-0.387141034	0.026545308	1.402442219	down
	Com_1941_neg	Flucythrinate	-0.864779437	0.02759117	3.801265254	down
	Com_6_neg	Myristic acid	0.312279586	0.033009041	1.111484155	up
	Com_2933_neg	Adinazolam	-0.354417627	0.036854922	1.296656541	down
	Com_1443_neg	1,3-Dihydroxy-2-propanyl 22-methyltetracosanoate	0.830033631	0.043467024	3.103481647	up
	Com_1199_neg	4-Formyl-2-methoxyphenyl hydrogen sulfate	-0.268141396	0.04909995	1.017529117	down
	Com_346_neg	Xanthine	-0.264866985	0.049398352	1.00265986	down
	Com_4419_neg	N-[(3a,5b,7b)-7-hydroxy-24-oxo-3-(sulfooxy)cholan-24-yl]-Glycine	-0.465329678	0.049944735	1.761267782	down
po_SI *vs* po_SINC	Com_7786_pos	GLY-MET	0.673865954	0.001806334	3.039279396	up
	Com_3355_pos	Leukotriene B4	0.58601866	0.001950563	2.765335181	up
	Com_2767_pos	13-Hydroxykaur-16-en-18-oic acid	0.537674459	0.002002927	2.462421819	up
	Com_5995_pos	L-(-)-Threonine	0.37237633	0.002118332	1.752482235	up
	Com_2404_pos	trimethadione	-0.474369385	0.00286851	2.231658333	down
	Com_6537_pos	2,3,4,5-tetrahydrodipicolinic acid	0.3647334	0.003313872	1.750164366	up
	Com_3572_pos	N-acetyl-L-2-aminoadipic acid	-0.385836722	0.004853561	1.771569865	down
	Com_968_pos	LysoPC(20:5(5Z,8Z,11Z,14Z,17Z))	-0.329572318	0.006098146	1.564981347	down

Continued table S1

Com_7935_pos	Atagabalin	0.437382724	0.008054952	2.075699288	up
Com_2145_pos	gitogenin	0.409146554	0.009712166	1.852558023	up
Com_1819_pos	Deoxyuridine monophosphate	0.39832963	0.012138553	1.897630652	up
Com_5269_pos	Nifedipine	0.302847172	0.013069161	1.415987256	up
Com_3917_pos	1-hexadecanoyl-sn-glycero-3-phosphoethanolamine	0.339068777	0.013110214	1.65668812	up
Com_7860_pos	(E)-1-methyldisulfanyl-3-methylsulfinyl-prop-1-ene	0.324360905	0.01399377	1.498969897	up
Com_3223_pos	Oxatomide	0.469485168	0.014627266	2.103375578	up
Com_5035_pos	L-Isoleucyl-L-alanyl-D-arginine	0.361520747	0.020688754	1.598582758	up
Com_128_pos	1-(beta-D-ribofuranosyl)thymine	0.284032677	0.021364416	1.369445784	up
Com_2724_pos	Heptanophenone	0.305134623	0.025909323	1.392366969	up
Com_792_pos	OLOPATADINE N-OXIDE	-0.331608864	0.026031237	1.601067494	down
Com_5519_pos	(3E,5Z)-6-(4-Chlorophenyl)-6-hydroxy-2-oxo-3,5-hexadienoic acid	0.383890589	0.028364445	1.899376662	up
Com_7767_pos	pyrazinamide	0.356815232	0.029334376	1.561603246	up
Com_6811_pos	Serotonin	0.268237846	0.030326101	1.297440596	up
Com_1488_pos	Thioperamide	0.505427679	0.030846787	2.193954315	up
Com_6475_pos	DDNU (VAN)	0.363909236	0.031429978	1.635648855	up
Com_9167_pos	1-(3-Phenyl-1H-1,2,4-triazol-5-yl)urea	0.331605069	0.033609763	1.588063515	up
Com_7968_pos	()-Camphoric acid	0.292426157	0.035953494	1.301684691	up
Com_1343_pos	2IAP3WIO1P	0.297073446	0.037829681	1.329859678	up

Continued table S1

	Com_5207_pos	Oleic acid	0.271873164	0.0386464	1.298990748	up
	Com_4867_pos	oxybutynin	0.265150987	0.038763929	1.305305561	up
	Com_8665_pos	Ethyl lactate	-0.301462425	0.041582208	1.388613554	down
	Com_7010_pos	Dibenzothiophene sulfone	0.320092412	0.043859555	1.607686713	up
	Com_8475_pos	Mannitol 1-phosphate	0.397944873	0.044207273	1.769032334	up
	Com_2926_pos	chloromebuform	-0.343103194	0.046720135	1.594498611	down
	Com_8466_pos	noradrenaline	0.482150963	0.047895529	2.102985501	up
	Com_5583_pos	Trimebutine	-0.380930455	0.048019813	1.778655825	down
	Com_3712_pos	Fingolimod	-0.510993477	0.048750851	2.567054067	down
	Com_5114_pos	(2R)-2-Hydroxy-3-(octadecyloxy)propyl dihydrogen phosphate	0.348201745	0.04881078	1.513772186	up
	Com_2265_neg	2-Naphthalenesulfonic acid	-0.661073821	0.002388547	1.72746783	down
	Com_3478_neg	carglumic acid	-0.384057013	0.003265781	1.021833275	down
	Com_3754_neg	N-Nitrosoproline	-0.440314747	0.008448882	1.078529461	down
	Com_2567_neg	(-)-Prostaglandin E2	1.697415707	0.017581893	3.824053782	up
	Com_2080_neg	actodigin	-0.488965995	0.023787986	1.330594721	down
	Com_3400_neg	EPTC	-0.57887019	0.025364776	1.805765356	down
	Com_1492_neg	Abietic acid	-1.253475051	0.048902345	2.443596827	down
