# Supplementary material for: Integrated Transcriptome and Metabolome Analysis Reveals the Regulatory Mechanisms of FASN in Geese Granulosa Cells
Source: Int J Mol Sci. 2022 Nov 25;23(23):14717. doi: 10.3390/ijms232314717 (PMC9736573; doi:10.3390/ijms232314717)
Supplement: Supplementary file 1 [file ijms-23-14717-s001.zip › ijms-2032364-supplementary Table S2.pdf]

Table S2. The KEGG pathways enriched by the differential metabolites

Group	MapID	MapTitle	Pvalue
ph_OE *vs* ph_OENC	map00240	Pyrimidine metabolism	0.019932
	map00520	Amino sugar and nucleotide sugar metabolism	0.042017
	map00730	Thiamine metabolism	0.159667
	map00350	Tyrosine metabolism	0.298255
	map04080	Neuroactive ligand-receptor interaction	0.298255
	map02010	ABC transporters	0.329866
	map04974	Protein digestion and absorption	0.329866
	map05204	Chemical carcinogenesis	0.058824
	map00250	Alanine, aspartate and glutamate metabolism	0.114656
	map00590	Arachidonic acid metabolism	0.114656
	map00983	Drug metabolism - other enzymes	0.114656
	map04664	Fc epsilon RI signaling pathway	0.114656
	map00730	Thiamine metabolism	0.217855
	map00400	Phenylalanine, tyrosine and tryptophan biosynthesis	0.265464
	map04080	Neuroactive ligand-receptor interaction	0.393695
	map02010	ABC transporters	0.431931
	map00232	Caffeine metabolism	0.06298
	map00230	Purine metabolism	0.093702
	map00061	Fatty acid biosynthesis	0.15361
ph_SI *vs* ph_SINC	map00780	Biotin metabolism	0.015873016
	map04977	Vitamin digestion and absorption	0.063492063
po_SI *vs* po_SINC	map04726	Serotonergic synapse	0.001424
	map04080	Neuroactive ligand-receptor interaction	0.001915
	map04540	Gap junction	0.0042
	map04742	Taste transduction	0.0042
	map04750	Inflammatory mediator regulation of TRP channels	0.0042
	map04024	cAMP signaling pathway	0.008256
	map04721	Synaptic vesicle cycle	0.008256
	map03320	PPAR signaling pathway	0.042017
	map04261	Adrenergic signaling in cardiomyocytes	0.042017
	map04270	Vascular smooth muscle contraction	0.042017
	map04924	Renin secretion	0.042017
	map04970	Salivary secretion	0.042017
	map04976	Bile secretion	0.05572

Continued table S2

	map00590	Arachidonic acid metabolism	0.082609
	map01040	Biosynthesis of unsaturated fatty acids	0.082609
	map04212	Longevity regulating pathway - worm	0.082609
	map04923	Regulation of lipolysis in adipocytes	0.082609
	map00061	Fatty acid biosynthesis	0.121814
	map01523	Antifolate resistance	0.121814
	map00240	Pyrimidine metabolism	0.231457
	map00350	Tyrosine metabolism	0.298255
	map00380	Tryptophan metabolism	0.298255
	map00340	Histidine metabolism	0.093702
