# Supplementary material for: Integrated Transcriptome and Metabolome Analysis Reveals the Regulatory Mechanisms of FASN in Geese Granulosa Cells
Source: Int J Mol Sci. 2022 Nov 25;23(23):14717. doi: 10.3390/ijms232314717 (PMC9736573; doi:10.3390/ijms232314717)
Supplement: Supplementary file 1 [file ijms-23-14717-s001.zip › ijms-2032364-supplementary Table S3.pdf]

Table S3. The top 20 GO terms enriched by DEGs in the four groups

Group	Category	GO	P-value	Gene name
ph_OE *vs* ph_OENC	BP	DNA integration	1.18E-07	LOC106035159/LOC106033736/LOC106035687/LOC106043734/LOC106036581/LOC106039938/-/LOC106034894/-/LOC106040511/LOC106044458
	MF	RNA-DNA hybrid ribonuclease activity	2.19E-07	LOC106035159/-/-/LOC106033736/LOC106035687/LOC106043734/LOC106036581/LOC106039938/LOC106034894/LOC106040511/LOC106044458
	MF	cytokine receptor binding	1.13E-06	LOC106041042/LOC106045177/LOC106041919/LOC106044139/LOC106048600/LOC106041921/LOC106048595/LOC106045178/IL6/LOC106041492/CCL20
	MF	endoribonuclease activity, producing 5'-phosphomonoesters	1.13E-06	LOC106035159/-/-/LOC106033736/LOC106035687/LOC106043734/LOC106036581/LOC106039938/LOC106034894/LOC106040511/LOC106044458
	MF	endonuclease activity, active with either ribo- or deoxyribonucleic acids and producing 5'-phosphomonoesters	1.63E-06	LOC106035159/-/-/LOC106033736/LOC106035687/LOC106043734/LOC106036581/LOC106039938/LOC106034894/LOC106040511/LOC106044458
	CC	extracellular region	2.30E-06	LOC106041042/THBS2/THBS1/LOC106045177/LOC106041919/LOC106039183/SPOCK1/MMP17/LOC106034120/LOC106044139/STC2/MFAP5/LOC106048600/LOC106041921/LOC106048595/PRRG4/LOC106048077/MMP7/LOC106045178/LOC106034204/IL6/GCG/WNT8A/LOC106041492/LOC106048082/LOC106034203/LOC106038135/CCL20
	MF	endoribonuclease activity	3.24E-06	LOC106035159/-/-/LOC106033736/LOC106035687/LOC106043734/LOC106036581/LOC106039938/LOC106034894/LOC106040511/LOC106044458

Continued table S3

	MF	G-protein coupled receptor binding	3.88E-06	LOC106041042/LOC106041919/LOC106048600/LOC106041921/LOC106048595/LOC106041492/CCL20
	MF	chemokine activity	3.88E-06	LOC106041042/LOC106041919/LOC106048600/LOC106041921/LOC106048595/LOC106041492/CCL20
	MF	chemokine receptor binding	3.88E-06	LOC106041042/LOC106041919/LOC106048600/LOC106041921/LOC106048595/LOC106041492/CCL20
	BP	apoptotic process	5.68E-06	BIRC2/ETV7/BID/BMF/LOC106040060/BAK1/BCL2L15/FADD/PMAIP1/CARD11/BCL2A1/BCL2L14
	BP	cell death	5.68E-06	BIRC2/ETV7/BID/BMF/LOC106040060/BAK1/BCL2L15/FADD/PMAIP1/CARD11/BCL2A1/BCL2L14
	BP	programmed cell death	5.68E-06	BIRC2/ETV7/BID/BMF/LOC106040060/BAK1/BCL2L15/FADD/PMAIP1/CARD11/BCL2A1/BCL2L14
	MF	endonuclease activity	8.56E-06	LOC106035159/-/-/LOC106033736/LOC106035687/LOC106043734/LOC106036581/ENDOV/LOC106039938/LOC106034894/LOC106040511/LOC106044458
	BP	regulation of cell death	9.55E-06	BIRC2/ETV7/BID/LOC106040060/BAK1/BCL2L15/FADD/PMAIP1/CARD11/BCL2A1/BCL2L14
	BP	regulation of apoptotic process	9.55E-06	BIRC2/ETV7/BID/LOC106040060/BAK1/BCL2L15/FADD/PMAIP1/CARD11/BCL2A1/BCL2L14
	BP	regulation of programmed cell death	9.55E-06	BIRC2/ETV7/BID/LOC106040060/BAK1/BCL2L15/FADD/PMAIP1/CARD11/BCL2A1/BCL2L14
	MF	ribonuclease activity	2.42E-05	LOC106035159/-/-/LOC106033736/LOC106035687/LOC106043734/LOC106036581/LOC106039938/LOC106034894/LOC106040511/LOC106044458

Continued table S3

	MF	nuclease activity	7.07E-05	LOC106035159/GEN1/-/-/LOC106033736/LOC106035687/LOC106043734/LOC106036581/ENDOV/LOC106039938/LOC106034894/EXO1/LOC106040511/LOC106044458
	MF	cytokine activity	8.25E-05	LOC106041042/LOC106041919/LOC106048600/LOC106041921/LOC106048595/LOC106041492/CCL20
ph_SI *vs* ph_SINC	MF	G-protein coupled receptor activity	0.002166209	HTR1B/ADGRD2/NPY2R/GPR139/ADRA2C/EDNRA/LOC106029414/DRD3/RXFP3
	MF	metal ion transmembrane transporter activity	0.002741073	SLC6A6/KCNE4/NIPAL4/SLC6A15/SCNN1A
	MF	oxidoreductase activity, acting on paired donors, with incorporation or reduction of molecular oxygen	0.00416652	LOC106033779/MOXD1/HMOX1/LOC106033102
	MF	sodium ion transmembrane transporter activity	0.005067435	SLC6A6/SLC6A15/SCNN1A
	MF	hyaluronic acid binding	0.005088002	CD44/STAB2
	MF	transmembrane signaling receptor activity	0.009082953	HTR1B/ADGRD2/NPY2R/GPR139/ADRA2C/EDNRA/LOC106029414/DRD3/RXFP3
	MF	signaling receptor activity	0.009616941	HTR1B/ADGRD2/NPY2R/GPR139/ADRA2C/EDNRA/LOC106029414/DRD3/RXFP3
	MF	glycosaminoglycan binding	0.01	CD44/STAB2
	MF	neurotransmitter transporter activity	0.011456	SLC6A6/SLC6A15
	MF	neurotransmitter:sodium symporter activity	0.011456	SLC6A6/SLC6A15

Continued table S3

	MF	solute:sodium symporter activity	0.011456329	SLC6A6/SLC6A15
	MF	inorganic cation transmembrane transporter activity	0.011977439	SLC6A6/KCNE4/NIPAL4/SLC6A15/SCNN1A
	BP	G-protein coupled receptor signaling pathway	0.01673832	HTR1B/ADGRD2/LOC106042779/NPY2R/GPR139/ADRA2C/EDNRA/LOC106029414/DRD3/RXFP3
	MF	signal transducer activity	0.016906908	HTR1B/ADGRD2/NPY2R/GPR139/ADRA2C/EDNRA/LOC106029414/DRD3/RXFP3
	MF	solute:cation symporter activity	0.018134918	SLC6A6/SLC6A15
	MF	transmembrane receptor activity	0.019545802	HTR1B/ADGRD2/NPY2R/GPR139/ADRA2C/EDNRA/LOC106029414/DRD3/RXFP3
	MF	monovalent inorganic cation transmembrane transporter activity	0.020381853	SLC6A6/KCNE4/SLC6A15/SCNN1A
	BP	cell adhesion	0.020630183	THBS3/CD44/LOC106049646/CDH18/STAB2
	BP	biological adhesion	0.020630183	THBS3/CD44/LOC106049646/CDH18/STAB2
	MF	cation transmembrane transporter activity	0.026362946	SLC6A6/KCNE4/NIPAL4/SLC6A15/SCNN1A
po_OE *vs* po_OENC	BP	immune system process	8.89E-05	LOC106041042/LOC106041919/LOC106041918/CX3CL1/TMEM173/CD74/RAG1
	MF	cytokine receptor binding	0.000234479	LOC106041042/LOC106045177/LOC106044139/LOC106041919/LOC106041918/CX3CL1
	MF	G-protein coupled receptor binding	0.000305338	LOC106041042/LOC106041919/LOC106041918/CX3CL1

Continued table S3

	MF	chemokine activity	0.000305338	LOC106041042/LOC106041919/LOC106041918/CX3CL1
	MF	chemokine receptor binding	0.000305338	LOC106041042/LOC106041919/LOC106041918/CX3CL1
	BP	immune response	0.000521672	LOC106041042/LOC106041919/LOC106041918/CX3CL1/TMEM173/CD74
	MF	NAD+ ADP-ribosyltransferase activity	0.001139082	LOC106031049/LOC106039304/PARP9/LOC106029916
	BP	defense response	0.001972106	LOC106045177/LOC106044139/TMEM173
	MF	cytokine activity	0.002363941	LOC106041042/LOC106041919/LOC106041918/CX3CL1
	MF	cation channel activity	0.00263593	TMEM37/LOC106029484/RYR2/KCNB1/KCNJ11/LOC106029719/KCNA4
	MF	regulatory region DNA binding	0.004670411	IRF7/IRF1/IRF8
	MF	regulatory region nucleic acid binding	0.004670411	IRF7/IRF1/IRF8
	MF	transferase activity, transferring pentosyl groups	0.006018761	LOC106031049/LOC106039304/PARP9/LOC106029916
	MF	metal ion transmembrane transporter activity	0.00948512	TMEM37/LOC106030949/LOC106029484/RYR2/KCNB1/KCNJ11/LOC106029719/KCNA4
	BP	monovalent inorganic cation transport	0.016590856	LOC106029484/KCNB1/KCNJ11/ATP6V0A4/LOC106029719/KCNA4
	BP	intracellular signal transduction	0.01673433	PRKD1/PLCD1/SIPA1L2/OBSCN/ARHGEF39/STAC/ASB15/LOC106044837/RASA3/RALGDS/ARHGEF9/ASB11
	BP	regulation of cell death	0.018029317	BIRC2/ETV7/BAK1/PMAIP1
	BP	regulation of apoptotic process	0.018029317	BIRC2/ETV7/BAK1/PMAIP1
	BP	regulation of programmed cell death	0.018029317	BIRC2/ETV7/BAK1/PMAIP1
	BP	metal ion transport	0.020834923	LOC106029484/RYR2/KCNB1/KCNJ11/LOC106029719/KCNA4

Continued table S3

po_SI *vs* po_SINC	MF	growth factor activity	0.00036237	CSF1/FGF12/LOC106037882/LOC106044377
	MF	G-protein coupled receptor activity	0.001197108	LOC106045532/HTR1B/HTR4/LOC106044607/CRHR2/GPR18/LOC106029318
	MF	protein heterodimerization activity	0.002948841	LOC106045907/LOC106045128/LOC106045148
	BP	phospholipid catabolic process	0.00325415	PLA2G4F/LOC106043002
	MF	transmembrane signaling receptor activity	0.003565767	LOC106045532/HTR1B/HTR4/LOC106044607/CRHR2/GPR18/LOC106029318
	MF	signaling receptor activity	0.003769187	LOC106045532/HTR1B/HTR4/LOC106044607/CRHR2/GPR18/LOC106029318
	MF	signal transducer activity	0.006534778	LOC106045532/HTR1B/HTR4/LOC106044607/CRHR2/GPR18/LOC106029318
	BP	cellular lipid catabolic process	0.006924449	PLA2G4F/LOC106043002
	BP	G-protein coupled receptor signaling pathway	0.006940596	LOC106045532/HTR1B/HTR4/LOC106044607/CRHR2/GPR18/LOC106029318
	MF	transmembrane receptor activity	0.007417778	LOC106045532/HTR1B/HTR4/LOC106044607/CRHR2/GPR18/LOC106029318
	BP	organophosphate catabolic process	0.008740864	PLA2G4F/LOC106043002
	BP	cell adhesion	0.010126262	HAPLN1/SPP1/PCDH15/RET
	BP	biological adhesion	0.010126262	HAPLN1/SPP1/PCDH15/RET
	BP	single-multicellular organism process	0.010153166	SPP1/JAG1/JAG2

Continued table S3

BP	anatomical structure development	0.010153166	PALMD/JAG1/JAG2
BP	developmental process	0.01064898	PALMD/JAG1/JAG2
BP	single-organism developmental process	0.01064898	PALMD/JAG1/JAG2
MF	receptor activity	0.012026754	LOC106045532/HTR1B/HTR4/LOC106044607/CRHR2/GPR18/LOC106029318
MF	molecular transducer activity	0.012026754	LOC106045532/HTR1B/HTR4/LOC106044607/CRHR2/GPR18/LOC106029318
BP	multicellular organismal process	0.013338085	SPP1/JAG1/JAG2
