# Supplementary material for: Integrated Transcriptome and Metabolome Analysis Reveals the Regulatory Mechanisms of FASN in Geese Granulosa Cells
Source: Int J Mol Sci. 2022 Nov 25;23(23):14717. doi: 10.3390/ijms232314717 (PMC9736573; doi:10.3390/ijms232314717)
Supplement: Supplementary file 1 [file ijms-23-14717-s001.zip › ijms-2032364-supplementary Table S4.pdf]

Table S4. The top 20 KEGG pathways enriched by DEGs in the four groups

Group	KEGG	P-Value	Gene Name
ph_OE *vs* ph_OENC	Herpes simplex infection	5.46E-06	STAT1/EIF2AK2/TLR3/IRF7/IFIH1/PML/LOC106049848/LOC106029502/TAP2/LOC106049847/LOC106049017/LOC106029505/IKBKE/LOC106045177/-/FADD/LOC106044208/LOC106049777/TRAF5/IL6/LOC106041040
	Influenza A	1.29E-05	HSPA2/STAT1/TRIM25/RSAD2/EIF2AK2/TLR3/IRF7/IFIH1/PML/LOC106036132/ADAR/LOC106049017/IKBKE/LOC106045177/LOC106041919/TMPRSS2/LOC106044208/IL6/LOC106041040
	NOD-like receptor signaling pathway	8.71E-05	STAT1/IRF7/BIRC2/LOC106029457/LOC106049017/IKBKE/LOC106046397/NAMPT/LOC106045177/LOC106041919/FADD/TNFAIP3/TMEM173/TRAF5/IL6/LOC106041040/PLCB4
	Cytokine-cytokine receptor interaction	0.0001	GHR/IL13RA1/LOC106045177/LOC106041919/PDGFRB/ACKR3/LOC106048600/TNFRSF9/CXCR4/CD40/LOC106048595/TNFSF4/CNTFR/TNFRSF11B/IL5RA/IL6/IL15RA/LOC106041040/IL12RB2/LOC106030637/CCL20
	RIG-I-like receptor signaling pathway	0.000244	TRIM25/LOC106047988/IRF7/IFIH1/IKBKE/LOC106045177/LOC106041919/LOC106042201/FADD/TMEM173
	Cytosolic DNA-sensing pathway	0.000296	IRF7/ADAR/IKBKE/LOC106045177/MB21D1/TMEM173/IL6/LOC106041040
	Cell adhesion molecules (CAMs)	0.000622	LOC106049848/LOC106049847/VCAN/LOC106029505/VCAM1/LOC106046390/CNTN1/CD40/LOC106041602/LRRC4C/LRRC4/CLDN10/VTCN1/LOC106043419
	Calcium signaling pathway	0.000665	ITPKA/LOC106045450/NOS2/BDKRB2/AGTR1/PLCD1/HRH1/PDGFRB/DRD1/HTR7/HTR6/ADRA1A/HTR4/CHRM3/PLCB4/LOC106029389/LOC106029839/BDKRB1/LOC106048195
	Toll-like receptor signaling pathway	0.00193	STAT1/TLR3/IRF7/IKBKE/LOC106045177/LOC106041919/MAP3K8/FADD/CD40/IL6/LOC106041040
	Neuroactive ligand-receptor interaction	0.005541	GHR/BDKRB2/AGTR1/PTH1R/GABRA4/HRH1/LPAR6/LOC106046928/DRD1/SSTR5/LOC106045532/HTR7/HTR6/GRID1/ADRA1A/HTR4/CHRM3/ADRA2C/LOC106029839/GALR3/GRM7/BDKRB1

Continued table S4

	Intestinal immune network for IgA production	0.007597	MAP3K14/CXCR4/CD40/IL6/IL15RA
	Necroptosis	0.016054	STAT1/EIF2AK2/TLR3/BIRC2/BID/LOC106049017/LOC106045177/LOC106042201/FADD/TNFAIP3/TRAF5/STAT4
	AGE-RAGE signaling pathway in diabetic complications	0.023429	STAT1/VCAM1/LOC106041919/AGTR1/EGR1/PLCD1/LOC106041602/NOX4/IL6/PLCB4
	Regulation of actin cytoskeleton	0.045422	SCIN/PAK2/BDKRB2/ARPC1B/PAK6/FGF12/PDGFRB/ITGB4/APC2/RAC2/MYL10/CHRM3/ITGA10/BDKRB1/FGF7
	Phagosome	0.097995712	LOC106049848/LOC106029502/TAP2/LOC106049847/C1R/THBS2/CTSS/THBS1/LOC106029505/TUBB1
	ECM-receptor interaction	0.106166189	THBS2/THBS1/COL9A3/AGRN/ITGB4/GP5/ITGA10
	p53 signaling pathway	0.113411105	BID/THBS1/FAM76A/PMAIP1/RRM2/SERPINB5
	Apoptosis	0.127759644	BIRC2/BID/CTSS/MAP3K14/CASP7/BAK1/DDIT3/FADD/PMAIP1/BCL2A1
	Protein processing in endoplasmic reticulum	0.14410058	HSPA2/EIF2AK2/BAG2/-/UFD1L/BAK1/FBXO44/DDIT3/-/-/-/-
	ABC transporters	0.156216458	LOC106029502/TAP2/ABCC3/ABCB11
ph_SI *vs* ph_SINC	Neuroactive ligand-receptor interaction	0.017056	HTR1B/NPY2R/ADRA2C/EDNRA/DRD3/RXFP3
	Influenza A	0.020044	HSPA2/SOCS3/RSAD2/LOC106032954
	Steroid hormone biosynthesis	0.028336	LOC106033779/LOC106045741
	Ferroptosis	0.04402	MAP1LC3C/HMOX1

Continued table S4

Adipocytokine signaling pathway	0.114316646	SOCS3/-
Glycosphingolipid biosynthesis - ganglio series	0.125050864	ST6GALNAC5
ECM-receptor interaction	0.152335984	COL4A1/CD44
Cytokine-cytokine receptor interaction	0.16292517	LOC106041672/CXCL14/IL20RA
Linoleic acid metabolism	0.177483297	LOC106033102
alpha-Linolenic acid metabolism	0.177483297	LOC106033870
Histidine metabolism	0.185918359	HNMT
Porphyrin and chlorophyll metabolism	0.194268911	HMOX1
AGE-RAGE signaling pathway in diabetic complications	0.203669511	COL4A1/LOC106032954
Biosynthesis of unsaturated fatty acids	0.226842417	LOC106033870
Citrate cycle (TCA cycle)	0.242643225	OGDHL
Nicotinate and nicotinamide metabolism	0.242643225	NMNAT2
Focal adhesion	0.253021136	COL4A1/-/LOC106032954
Alanine, aspartate and glutamate metabolism	0.273304323	GFPT2

Continued table S4

	Apelin signaling pathway	0.27963351	PIK3R6/JAG1
	Tryptophan metabolism	0.295501142	OGDHL
po_OE *vs* po_OENC	Herpes simplex infection	8.90E-10	STAT1/TLR3/EIF2AK2/IRF7/IFIH1/PML/LOC106049848/LOC106029502/TAP2/LOC106049017/IKBKE/LOC106049847/LOC106029505/LOC106045177/LOC106044208/LOC106044308/CD74/TRAF5
	Influenza A	1.41E-09	HSPA2/STAT1/TRIM25/TLR3/EIF2AK2/IRF7/IFIH1/ADAR/RSAD2/PML/LOC106036132/LOC106049017/IKBKE/LOC106045177/LOC106044208/LOC106041919/TMPRSS2
	NOD-like receptor signaling pathway	1.74E-05	STAT1/IRF7/BIRC2/LOC106049017/IKBKE/TNFAIP3/LOC106045177/LOC106046397/LOC106041919/LOC106029457/TMEM173/TRAF5
	RIG-I-like receptor signaling pathway	1.89E-05	TRIM25/LOC106047988/IRF7/IFIH1/IKBKE/LOC106045177/LOC106041919/TMEM173
	Cytosolic DNA-sensing pathway	0.000110768	IRF7/ADAR/IKBKE/LOC106045177/MB21D1/TMEM173
	Cytokine-cytokine receptor interaction	0.001184472	LOC106045177/AMH/LOC106041919/CX3CL1/IL21R/IFNLR1/IL12RB2/IL20RB/IL5RA/LOC106030637/BMP7
	Necroptosis	0.001631465	STAT1/TLR3/EIF2AK2/BIRC2/LOC106049017/TNFAIP3/LOC106045177/STAT4/TRAF5
	Toll-like receptor signaling pathway	0.002012945	STAT1/TLR3/IRF7/IKBKE/LOC106045177/MAP3K8/LOC106041919
	Pyrimidine metabolism	0.007070196	CMPK2/NT5C3B/LOC106037128/ENTPD3/DCTD/LOC106034733/UPP2
	Cell adhesion molecules (CAMs)	0.00851846	LOC106049848/VCAN/LOC106046390/LOC106049847/LOC106029505/LRRC4/LOC106049813
	Phagosome	0.023170375	LOC106049848/LOC106029502/TAP2/LOC106049847/LOC106029505/ATP6V0A4/LOC106030923

Continued table S4

	Cardiac muscle contraction	0.03310851	LOC106048992/MYL2/MYH7B/RYR2
	ABC transporters	0.065180502	LOC106029502/TAP2/ABCA12
	Calcium signaling pathway	0.176820999	ITPKA/PLCD1/DRD1/RYR2/LOC106029839/CHRM3
	Apoptosis	0.186879675	BIRC2/CASP7/BAK1/PMAIP1/LOC106030923
	Glycine, serine and threonine metabolism	0.229246919	PHGDH/LOC106036144
	Arginine and proline metabolism	0.25913539	AZIN2/LOC106047481
	Cellular senescence	0.26001821	LOC106049848/CDC25A/LOC106049847/LOC106029505/LOC106041919
	Non-homologous end-joining	0.277279475	DNTT
	Fatty acid biosynthesis	0.296604468	FASN
po_SI *vs* po_SINC	Linoleic acid metabolism	0.009145	PLA2G4F/LOC106043002
	alpha-Linolenic acid metabolism	0.011241	PLA2G4F/LOC106043002
	ABC transporters	0.030911	TAP2/ABCB4
	Ether lipid metabolism	0.036143	PLA2G4F/LOC106043002
	Notch signaling pathway	0.036143	JAG1/JAG2
	Arachidonic acid metabolism	0.039808	PLA2G4F/LOC106043002
	Apelin signaling pathway	0.047122	GNB3/SPP1/JAG1

Continued table S4

	Cardiac muscle contraction	0.05791065	ACTC1/LOC106032749
	VEGF signaling pathway	0.068990687	PLA2G4F/LOC106043002

Neuroactive ligand-receptor interaction	0.070376169	LOC106045532/HTR1B/HTR4/CRHR2
PPAR signaling pathway	0.073605902	CD36/LOC106029718
Phenylalanine metabolism	0.078889921	HPD
TGF-beta signaling pathway	0.098072	LOC106037882/LOC106044377
Glycosphingolipid biosynthesis - ganglio series	0.101355019	ST8SIA5
ECM-receptor interaction	0.103215247	SPP1/CD36
Glycosaminoglycan biosynthesis - keratan sulfate	0.108724515	CHST1
MAPK signaling pathway	0.114270127	PLA2G4F/CSF1/FGF12/LOC106043002
Phototransduction	0.116035339	GNB3
GnRH signaling pathway	0.127230208	PLA2G4F/LOC106043002
Glycerophospholipid metabolism	0.143922773	PLA2G4F/LOC106043002
