# Supplementary material for: Integrated Transcriptome and Metabolome Analysis Reveals the Regulatory Mechanisms of FASN in Geese Granulosa Cells
Source: Int J Mol Sci. 2022 Nov 25;23(23):14717. doi: 10.3390/ijms232314717 (PMC9736573; doi:10.3390/ijms232314717)
Supplement: Supplementary file 1 [file ijms-23-14717-s001.zip › ijms-2032364-supplementary Table S5.pdf]

Table S5. Hub genes in the networks ranked by Degree method

Group	Rank	Name	Score
ph_OE vs ph_OENC	1	STAT1	1405
	2	IRF7	1368
	3	TLR3	1330
	4	IFIH1	1038
	5	EIF2AK2	960
	6	RSAD2	840
	7	TRIM25	745
	8	IL6	524
	9	CD40	425
	10	CXCR4	390
	11	BDKRB1	264
	11	BDKRB2	264
	13	AGTR1	146
	14	STAT4	144
	14	GALR3	144
	14	SSTR5	144
	17	VCAM1	135
	18	ADAR	126
	19	HRH1	120
	19	ADRA2C	120
	19	CHRM3	120
	19	ADRA1A	120
	23	IKBKE	94
	24	TMEM173	48
	25	TNFAIP3	41
	26	MB21D1	32
	27	HTR6	24
	27	HTR4	24
	27	HTR7	24
	27	DRD1	24
	27	PTH1R	24
	32	FADD	21
	33	BIRC2	12
	34	PDGFRB	8
	34	TRAF5	8
	36	NOS2	6
	37	MAP3K14	4
	37	NOX4	4
	39	RAC2	3
	39	NAMPT	3
	41	MAP3K8	2

Continued table S5

	41	FGF7	2
	41	PLCB4	2
	41	ITPKA	2
	41	TNFRSF9	2
	41	PLCD1	2
	41	FGF12	2
	48	EGR1	1
	48	PAK2	1
	48	VCAN	1
	48	IL13RA1	1
	48	PAK6	1
	48	BID	1
	48	TNFRSF11B	1
	48	VTCN1	1
	48	HSPA2	1
	48	PML	1
po_OE vs po_OENC	1	STAT1	14
	2	IRF7	13
	3	TLR3	12
	4	IFIH1	11
	5	CMPK2	9
	5	RSAD2	9
	7	EIF2AK2	8
	8	IKBKE	7
	8	STAT4	7
	8	TRIM25	7
	11	ADAR	6
	12	TNFAIP3	5
	12	TMEM173	5
	12	MB21D1	5
	15	NT5C3L	4
	16	IL12RB2	3
	16	IFNLR1	3
	16	DCTD	3
	16	BIRC2	3
	20	MYL2	2
	20	IL21R	2
	20	ENTPD3	2
	20	UPP2	2
	20	TRAF5	2
	25	MYH7B	1
	25	IL20RB	1

Continued table S5

	25	RYR2	1
	25	MAP3K8	1
	25	IL5RA	1
	25	PML	1
